# Comparison of single taper and dual taper versions of the same stem design in total hip arthroplasty for primary osteoarthritis

**DOI:** 10.1186/s10195-023-00687-6

**Published:** 2023-02-01

**Authors:** Francesco Castagnini, Barbara Bordini, Monica Cosentino, Enrico Tassinari, Giulia Guizzardi, Francesco Traina

**Affiliations:** 1 Ortopedia-Traumatologia e Chirurgia Protesica e Dei Reimpianti d’anca E Di Ginocchio, IRCCS Istituto Ortopedico Rizzoli, Via Pupilli 1, 40136 Bologna, Italy; 2 Laboratorio Di Tecnologia Medica, IRCCS Istituto Ortopedico Rizzoli, Via Di Barbiano 1/10, 40136 Bologna, Italy; 3 DIBINEM, Università Di Bologna, Bologna, Italy

**Keywords:** Dual taper, Exchangeable neck, Dislocation, Corrosion, Failure

## Abstract

**Background:**

In total hip arthroplasty (THA), the outcomes of single taper (ST) and dual taper (DT) versions of the same stem design have been scarcely studied. A registry study comparing ST and DT versions of the same stem design was designed, aiming to assess: (1) the survival rates and the hazard ratios for failure; (2) the survival rates and the hazard ratios for failure using stem-focused endpoints.

**Material and methods:**

A regional arthroplasty registry was interrogated about stem designs with ST and DT versions in cementless THAs performed for primary osteoarthritis. Only the same cup and ceramic-on-ceramic bearings were included: the DT stems had a titanium-on-titanium modularity. Demographic and implant features were recorded. Survival rates and hazard ratios were evaluated and compared. Stem-focused endpoints were also investigated.

**Results:**

A total of 5359 THAs were included, with three stem designs. The two versions of every stem showed different demographics and implant-related features: ST versions were preferentially implanted in heavier young men. For each stem, the two versions had similar survival rates at 5 years (*p* = 0.076; *p* = 0.319; *p* = 0.616) and similar adjusted hazard ratios for failures (*p* = 0.084; *p* = 0.308; *p* = 0.729). When stem-focused endpoints were adopted, the ST and DT versions of the three stems achieved similar survival rates (*p* = 0.710; *p* = 0.784; *p* = 0.983) and similar adjusted hazard ratios (*p* = 0.647; *p* = 0.858; *p* = 0.787). Three neck breakages occurred (0.0007% of all the modular implants).

**Conclusions:**

ST and DT versions of the same stem design did not show any differences in terms of survival rates and hazard ratios for failures at 5 years.

*LEVEL OF EVIDENCE*: IV.

## Main text

### Introduction

Proximal femoral modularity in total hip arthroplasty (THA) aimed to restore the hip anatomy, improving hip biomechanics and implant stability [1]. The theoretical benefits of dual taper (DT) implants over single taper (ST) stems were evident in some radiographic studies: modularity was noticed to more closely reproduce the femoral morphologies, even in outlier anatomies [2, 3]. However, these benefits did not translate into higher survival rates, according to large database studies: on the contrary, modularity showed higher revision rates due to implant failures [4, 5]. Large database studies concluded that the routine use of exchangeable necks in THAs performed for primary osteoarthritis should be discouraged: even in case of compatible titanium alloy modularity, the higher revision rates outweighed the limited tangible benefits [4, 5].

However, it is acknowledged that the outcomes of DT implants are strongly dependent on the design of every femoral component [6]. Thus, to appreciate the different outcomes of ST and DT implants, the same stem designs with the DT and ST configurations should be compared. To date, there is a paucity of available literature comparing the same stem design in the ST and DT versions [3, 5, 7, 14].

Thus, a regional arthroplasty registry was interrogated about three titanium alloy stems with an ST and a DT version (titanium alloy exchangeable necks). Only THAs performed for primary osteoarthritis, with the same cup and the same bearing surfaces, were included. For every single design with ST and DT versions, we sought to assess and compare: (1) the survival rates and the adjusted hazard ratio (HR) for failures of the two versions; (2) the survival rates and the adjusted HR for stem-focused reasons for revisions (stem aseptic loosening, global aseptic loosening, primary instability/dislocations, implant breakage).

## Materials and methods

The regional arthroplasty registry RIPO provides active surveillance in the Italian region Emilia-Romagna (around 4,500,000 inhabitants), collecting data on hip, knee, and shoulder arthroplasties and revision surgeries since January 2000 [8]. Involving 68 orthopedic facilities in the region, the registry is a cross-checked database with a reported capture rate of 98%, with the 2% of missing data due to the lack of adherence [8]. RIPO collects the forms filled by all the surgeons performing primary arthroplasty or revision surgeries: the clinical conditions of the patients, the devices (batch and code), and the surgical technique (approach and fixation) are reported.

**Fig. 1:**
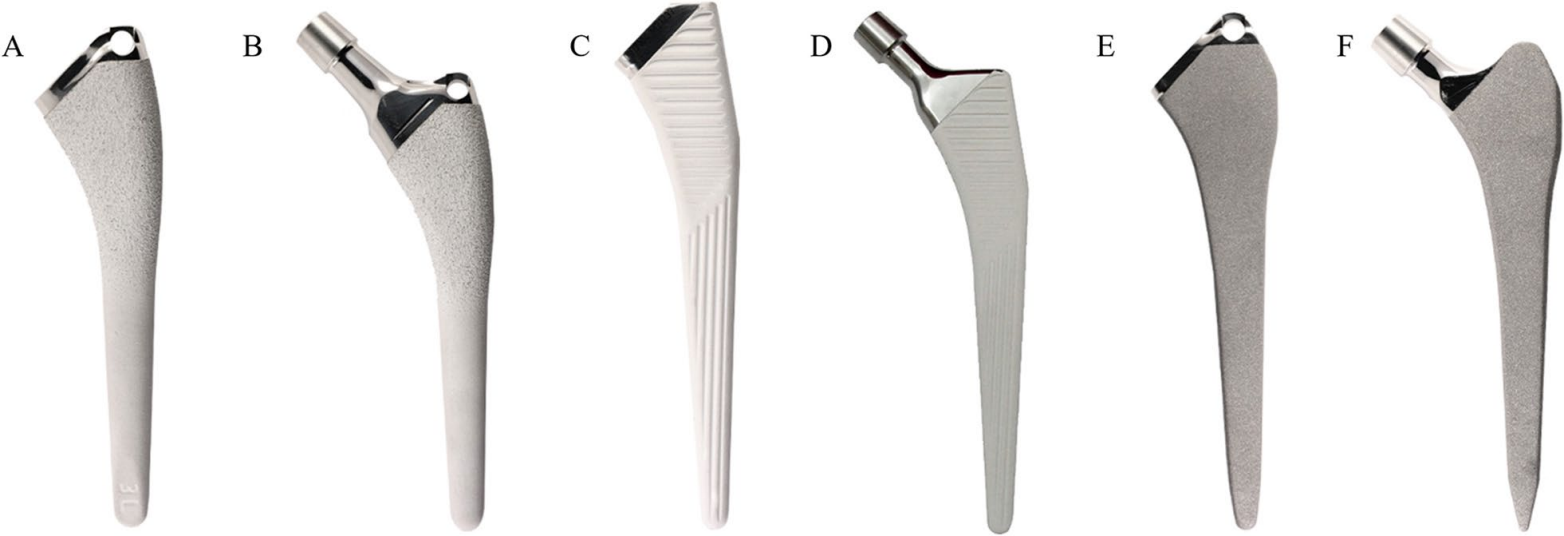
The six stems involved in the study were manufactured by Adler Ortho (Milan, Italy): Apta (**A**), Apta-fix (**B**), Hydra (**C**), Hydra-fix (**D**), Recta (**E**), and Recta-fix (**F**)

**Fig. 2:**
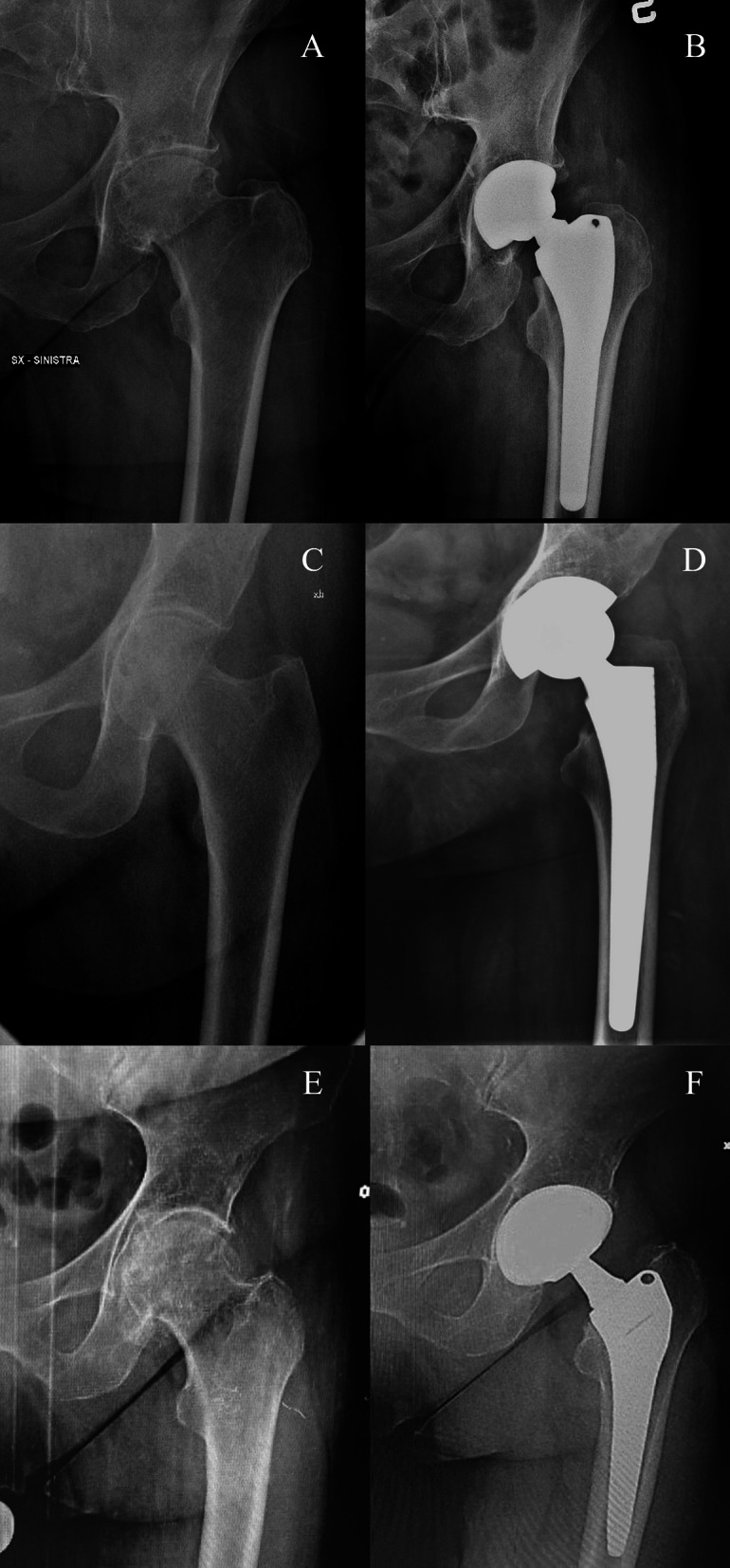
Preoperative (**A**, **C**, **E**) and postoperative (**B**, **D**, **F**) pelvis X-rays of three clinical cases, one for each stem with the DT version: Apta (**B**), Hydra (**D**), and Recta (**F**)

The RIPO registry was inquired about three stems implanted in primary cementless THAs with two versions, ST and DT. The three involved stems, with the ST and DT versions, were Apta-fix/Apta (Adler Ortho, Milan, Italy), Hydra-fix/Hydra (Adler Ortho, Milan, Italy), and Recta-fix/Recta (Adler Ortho, Milan, Italy) (Fig. 1). In the DT versions (Apta, Hydra, Recta), all the three stems shared the same titanium alloy Ti6Al4V and the same modular junction, Modula (Adler Ortho, Milan, Italy), providing 27 version/offset/length combinations by using 15 Ti6Al4V modular necks (Fig. 2).

Apta-fix design is an anatomic stem with an extensive hydroxyapatite coating (classification according to Khanuja (6): it is available in eight sizes, with standard and offset configurations (lateralization of 7.5 mm) [9, 10]. The caput-collum-diaphyseal (CCD) angle changes from the standard configuration (135°) to the offset one (130°) [9]. Both the standard and offset solutions have 6° of neck anteversion [9]. Hydra-fix design is a hydroxyapatite-coated single wedge tapered stem (classification according to Khanuja: 1): it is available in 11 sizes, with standard and offset configurations (lateralization of 7.5 mm) [10, 11]. Standard and offset configurations share the same CCD angle (135°) [11]. Recta-fix is a corundum blasted stem with a tapered rectangular design (classification according to Khanuja: 3C): nine sizes are available, with standard (CCD 131°) and offset configurations (CCD 123°, with 7.5 mm lateralization) [10, 12].

The inclusion criteria were: residing patients (to minimize the loss of patients at follow-up), THAs performed for primary osteoarthritis, use of Delta ceramic-on-Delta ceramic bearings (Ceramtec, Plochingen, Germany), THAs with 3D-printed Fixa TiPor (Adler) socket, modern version of titanium-on-titanium modular junctions (identified by “046XXXX” code).

All the THAs performed for reasons others than primary osteoarthritis, in nonresiding patients, with bearings others than Delta-on-Delta and cups others than TiPor Adler and involving previous versions of the modular junction were excluded.

Every selected design was stratified into two cohorts according to modularity, ST and DT: three pairs of cohorts were eventually identified. Demographics and implant-related features of ST and DT cohorts were collected and compared for every single design. The survival rates of ST and DT cohorts were calculated and compared, using different endpoints. Similarly, adjusted HRs for different reasons for failure were calculated and compared.

Institutional review board approval was waived due to the registry nature of the study and data anonymization.

### Statistical analysis

Statistical analyses were performed using SPSS software (version 14.0.1, Chicago, IL) JMP, version 12.0.1 (SAS Institute Inc, Cary, NC, 1989–2007. Data were provided as raw data, ranges, frequencies, and percentages. Continuous variables of demographic and implant-related features were analyzed using Student’s *t*-test, and frequencies and percentages using chi-square test. The survival curves were calculated and plotted using the Kaplan–Meier method (time in years on the *x*-axis and percentage of survived implants on the *y*-axis): the curve was flanked by a pair of 95% confidence interval curves. The implants were considered “surviving” at the last date of observation (date of death or 3 December 31, 2020) when no single component was replaced. The log-rank test was adopted to test the survival curves (threshold, *p* = 0.05). A multivariate Cox regression model was used to detect failures, with Wald test to detect any significance. HRs and 95% confidence intervals were specified. Threshold for significance was *p* = 0.05.

### Results

A total of 5789 THAs were included. By stratifying the implants per version, 1984 (75.9%) Apta and 629 (24.1%) Apta-fix; 1672 (70.8%) Hydra and 690 (29.2%) Hydra-fix; 662 (81.3%) Recta and 152 (18.7%) Recta-fix were enrolled. Apta, Hydra, and Recta (the three DT versions) had a female prevalence (respectively 64.5%, 66%, and 59.2%; *p* < 0.001). The mean age, mean height, and mean weight of the Apta/Apta-fix cohorts were, respectively, 69.6 years (range: 37–92 years) and 63.6 years (range: 24–96 years) (*p* < 0.001); 164.9 cm (range: 130–192 cm) and 171.9 cm (range: 149–200 cm) (*p* < 0.001); 73.6 kg (range: 38–170 kg) and 85 kg (range: 42–138 kg) (*p* < 0.001). The mean age, mean height, and mean weight of the Hydra/Hydra-fix cohorts were, respectively, 69.3 years (range: 34–90 years) and 67.7 years (range: 40–90 years) (*p* < 0.001); 164.6 cm (range: 140–195 cm) and 168.7 cm (range: 140–198 cm) (*p* < 0.001); 73 kg (range: 33–150 kg) and 81.4 kg (range: 45–125 kg) (*p* < 0.001). The mean age, mean height, and mean weight of the Recta/Recta-fix cohorts were, respectively, 69.1 years (range: 35–90 years) and 67.7 years (range: 44–85 years) (*p* = 0.087); 166.6 cm (range: 140–190 cm) and 167.9 cm (range: 140–190 cm) (*p* = 0.009); 75.6 kg (range: 42–120 kg) and 79.9 kg (range: 47–149 kg) (*p* < 0.001). The distribution per age decade and body mass index (BMI) class are detailed in the table: there was a significant difference in terms of age decade for Apta/Apta-fix and Hydra/Hydra-fix cohorts (*p* < 0.001) and for all the three pairs in terms of BMI class (Apta/Apta-fix, *p* < 0.001; Hydra/Hydra-fix, *p* < 0.001; Recta/Recta-fix, *p* = 0.04) (Table 1). The two pairs of cohorts Apta/Apta-fix and Hydra/Hydra-fix were not comparable in terms of head size (higher 36 mm rates in the ST cohorts, *p* < 0.001) and cup size (bigger sockets in ST cohorts, *p* < 0.001). Recta/Recta-fix cohorts were comparable in terms of head size (*p* = 0.091) and cup size (*p* = 0.316). The three pairs of stems were not comparable in terms of stem size (larger sizes in DT cohorts, *p* < 0.001).

**Table 1:** The distribution of the implants per age decade and per BMI class in ST and DT versions of all three pairs

	Apta		Apta-fix		Hydra		Hydra-fix		Recta		Recta-fix	
	*N*	%	*N*	%	*N*	%	*N*	%	*N*	%	*N*	%
Age per decade												
< 40	1	0.1	11	1.7	8	0.5	0	0	8	1.3	0	0
40–49	45	2.3	47	7.5	44	2.6	27	3.9	16	2.4	4	2.8
50–59	219	11	144	22.9	165	9.9	96	13.9	75	11.3	21	13.8
60–69	624	31.5	236	37.5	570	34.1	257	37.2	197	29.8	53	34.9
70–79	885	44.6	163	25.9	703	42.1	262	37.9	296	44.7	69	45.4
≥ 80	210	10.6	28	4.5	181	10.8	49	7.1	70	10.6	5	3.3
BMI class												
Underweight	10	0.5	0	0	9	0.5	3	0.4	4	0.6	0	0
Normal weight	645	32.3	124	19.7	352	21.1	103	14.9	229	34.6	61	40.1
Overweight	850	42.8	270	42.9	520	31.1	234	33.9	121	18.3	61	40.1
Obese	409	20.5	208	33.1	213	12.7	168	24.3	121	18.3	49	32.2
Missing data	70	3.5	27	4.3	577	34.5	183	26.5	147	22.2	8	5.3

### Survival rates and adjusted HR for failures

**Fig. 3:**
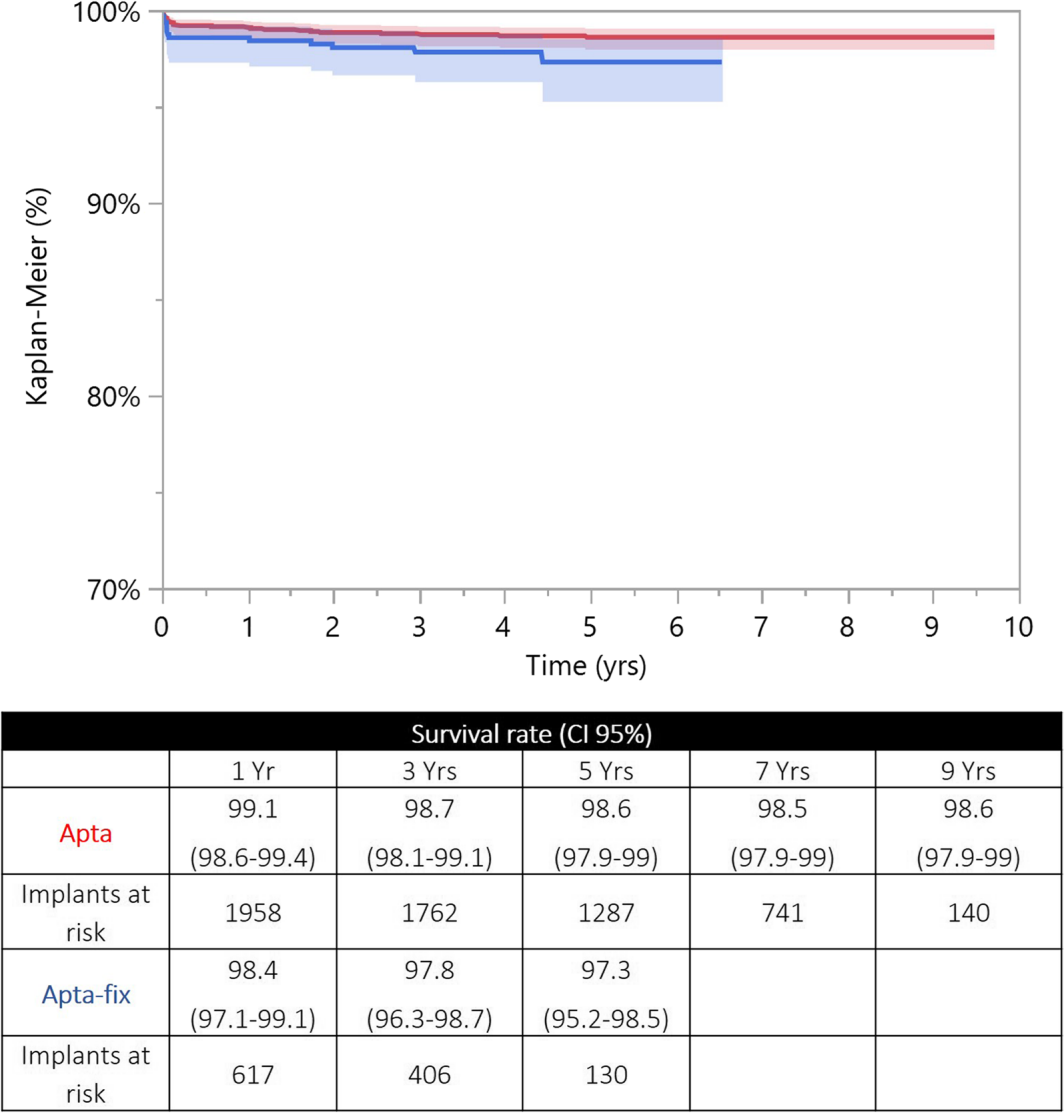
Kaplan-Meier curves of the Apta (in red) and Apta-fix (in blue) stems: the two versions achieved similar survival rates when the endpoint was revision for any reason. *y*-axis, percentage of survival implants; *x*-axis, years

**Fig. 4:**
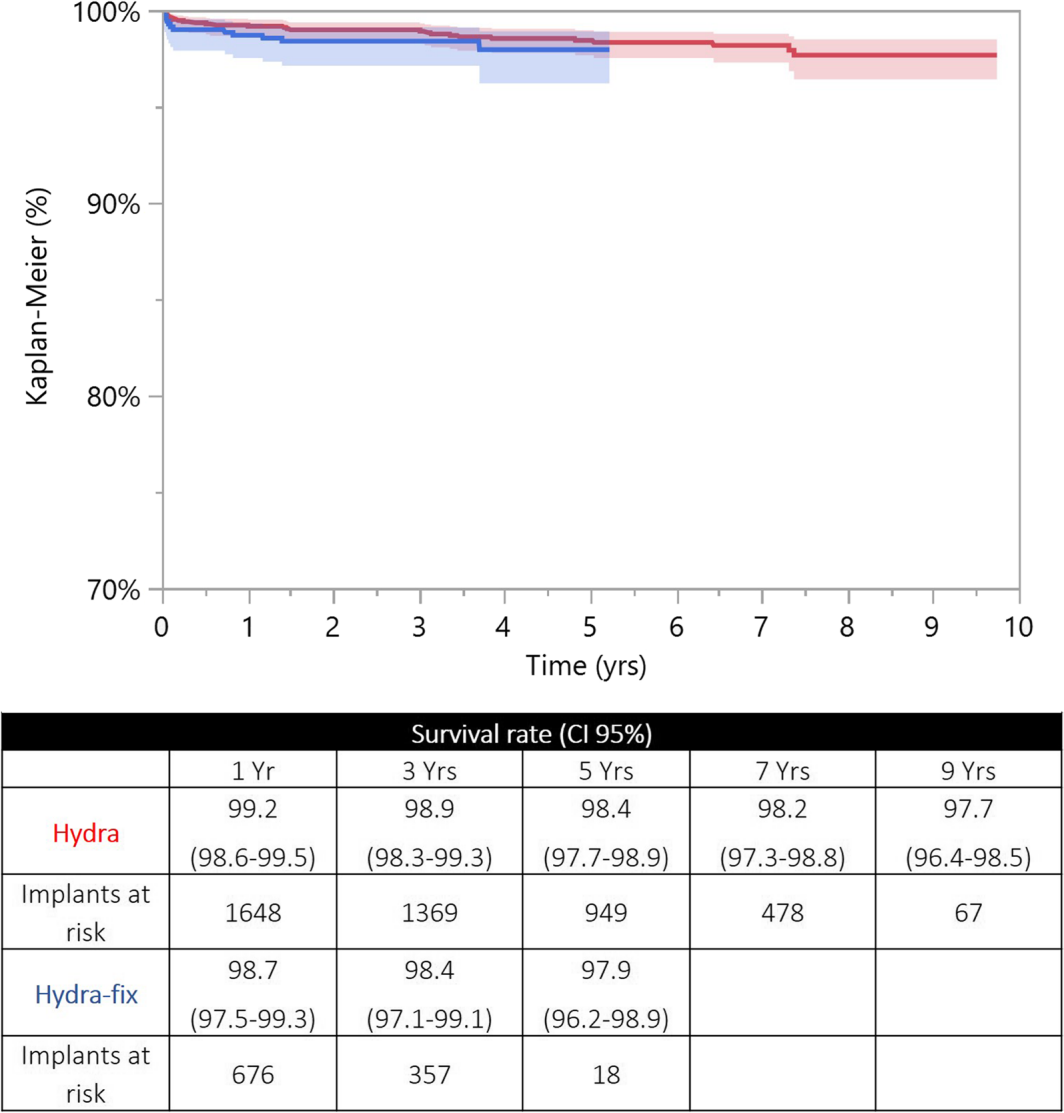
Kaplan–Meier curves of the Hydra (in red) and Hydra-fix (in blue) stems: the two versions achieved similar survival rates when the endpoint was revision for any reason. *y*-axis, percentage of survival implants; *x*-axis, years

**Fig. 5:**
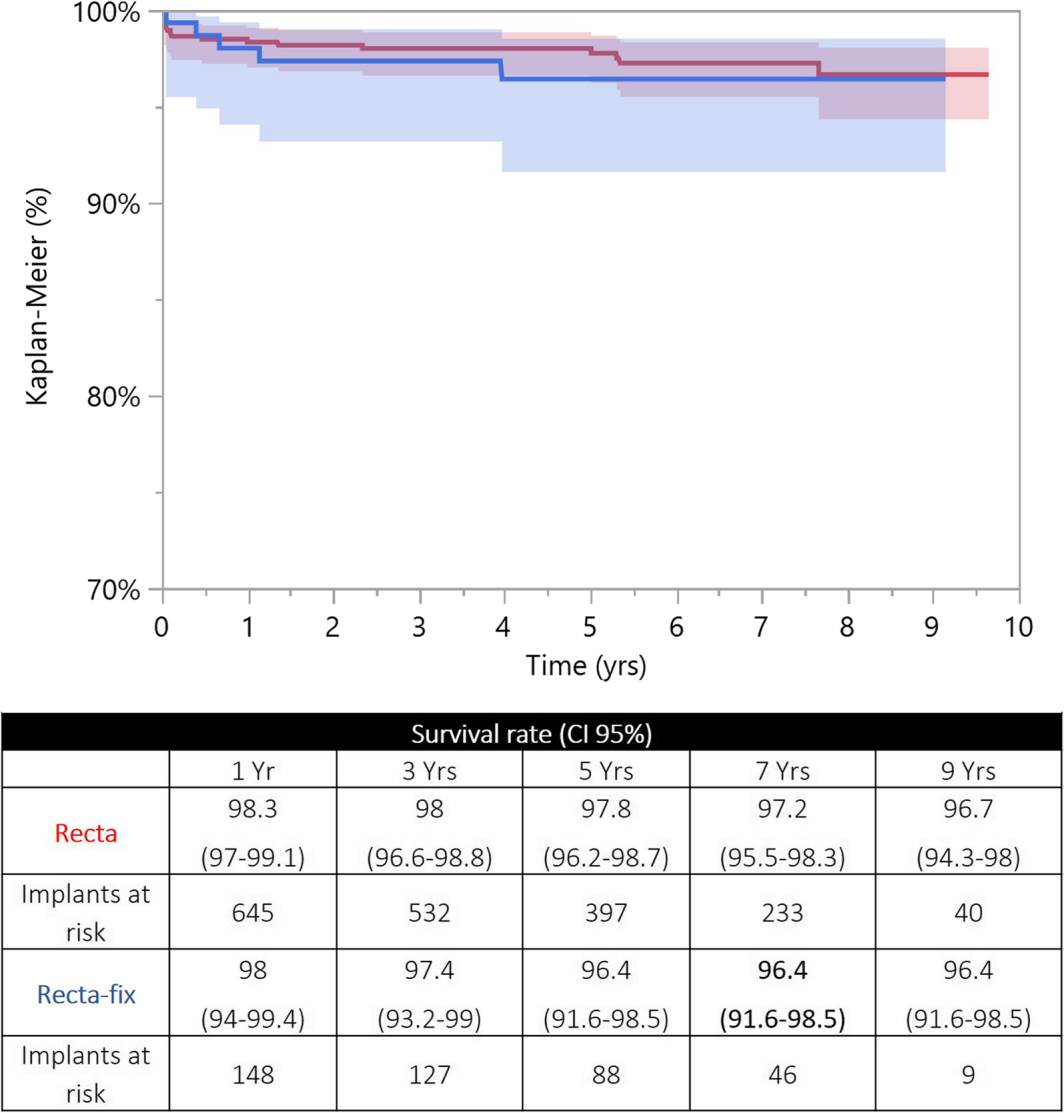
Kaplan–Meier curves of the Recta (in red) and Recta-fix (in blue) stems: the two versions achieved similar survival rates when the endpoint was revision for any reason. *y*-axis, percentage of survival implants; *x*-axis, years

For each stem design, the two curves were fully reliable at 5 years (> 10% of the implants at risk). At a mid-term follow-up, ST and DT versions achieved comparable survival rates (Apta/Apta-fix: *p* = 0.076; Hydra/Hydra-fix: *p* = 0.319; Recta/Recta-fix: *p* = 0.616) (Figs. 3, 4, 5). For each stem design, the HR adjusted for age (categorical variable, > 65 years or ≤ 65 years) and sex showed that the two versions were not different in terms of revisions (Apta/Apta-fix: *p* = 0.084; Hydra/Hydra-fix: *p* = 0.308; Recta/Recta-fix: *p* = 0.729). For each stem design, the reasons for revision of the two versions are reported in Table 2.

**Table 2:** Reasons for revision of the three pairs of cohorts were represented as incidence, percentage, and distribution of failures

Reasons for revision	Apta (1984)			Apta-fix (629)			Hydra (1671)			Hydra-fix (691)			Recta (662)			Recta-fix (152)		
	*N*	Incidence (%)	Frequency of failure (%)	*N*	Incidence (%)	Frequency of failure (%)	*N*	Incidence (%)	Frequency of failure (%)	*N*	Incidence (%)	Frequency of failure (%)	*N*	Incidence (%)	Frequency of failure (%)	*N*	Incidence (%)	Frequency of failure (%)
Pain without loosening	0	0	0	1	0.2	7.1	3	0.2	10.7	0	0	0	3	0.5	17.6	0	0	0
Periprosthetic fracture	5	0.3	18.5	5	0.8	35.7	7	0.4	25	3	0.4	25	0	0	0	0	0	0
Dislocation	8	0.4	29.6	0	0	0	2	0.1	7.1	1	0.1	8.3	3	0.5	17.6	1	0.7	20
Cup aseptic loosening	3	0.2	11.1	1	0.2	7.1	2	0.1	7.1	0	0	0	3	0.5	17.6	0	0	0
Global aseptic loosening	0	0	0	0	0	0	0	0	0	0	0	0	0	0	0	1	0.7	20
Stem aseptic loosening	1	0.1	3.7	1	0.2	7.1	3	0.2	10.7	1	0.1	8.3	1	0.2	5.9	0	0	0
Infection	2	0.1	7.4	1	0.2	7.1	2	0.1	7.1	1	0.1	8.3	0	0	0	0	0	0
Unknown	1	0.1	3.7	1	0.2	7.1	4	0.2	14.3	1	0.1	8.3	1	0.2	5.9	1	0.7	20
Ossifications	2	0.1	7.4	0	0	0	0	0	0	0	0	0	1	0.2	5.9	0	0	0
Primary instability	2	0.1	7.4	1	0.2	7.1	1	0.1	3.6	2	0.3	16.7	3	0.5	17.6	0	0	0
Neck breakage	2	0.1	7.4	0	0	0	0	0	0	0	0	0	1	0.2	5.9	0	0	0
Liner breakage	1	0.1	3.7	0	0	0	3	0.2	10.7	1	0.1	8.3	0	0	0	1	0.7	20
Stem breakage	0	0	0	0	0	0	1	0.1	3.6	0	0	0	0	0	0	1	0.7	20
Total	27	1.4	100	14	2.2	100	28	1.7	100	12	1.7	100	17	2.6	100	5	3.3	100

### Survival rates and adjusted HR for stem-focused reasons for revisions

When stem-focused endpoints were adopted, ST and DT versions of the three stems achieved similar survival rates at 5 years (Apta/Apta-fix: *p* = 0.710; Hydra/Hydra-fix: *p* = 0.784; Recta/Recta-fix: *p* = 0.983). For each stem, the HR adjusted for age and sex showed that the two versions were comparable in terms of revisions for stem and neck failures (Apta/Apta-fix: *p* = 0.647; Hydra/Hydra-fix: *p* = 0.858; Recta/Recta-fix: *p* = 0.787). Three neck breakages occurred (0.0007% of all the modular implants), two with Apta and one with Recta stems (all with 36 mm heads). The two neck failures in the Apta cohort occurred in obese men (BMI 31 and 36 kg/m^2^; weight > 90 kg); the patients were 67 years old and 72 years old. The breakages occurred 4.94 and 3.95 years after the first implant. Both the failures underwent neck exchange with no stem removal. The neck failure in the Recta cohort occurred in a 52-year-old man (BMI 27 kg/m^2^, weight 90 kg), 7.67 years after the first implant. In this case, a stem revision was performed.

## Discussion

For each stem design, ST and DT versions achieved similar survival curves and adjusted HRs for failure at 5 years. The survival rates and adjusted HRs were similar for both the versions even when stem-focused reasons for revision were considered. Neck failures occurred in only three cases (0.0007% of all the modular implants), all of whom were men weighting ≥ 90 kg.

This study has some limitations due to the registry nature. Registries cannot provide those clinical and radiological outcomes that would be necessary to demonstrate the theoretical benefits of DT implants in terms of biomechanics restoration and improved implant stability [1, 8]. Moreover, patient-reported outcomes were not reported. Even in some titanium alloy modular junctions, the issue of ion release and possible generation of adverse reactions to metal debris may occur: registries do not provide data about such complications unless revision is performed [3, 8]. Other relevant limits are the noncomparable demographic and implant-related features in the two versions, preventing a perfect comparison between the two cohorts. However, the recent literature discourages modular implants in active, heavy, and young men, inevitably leading to two different spectra of target patients for ST and DT versions [4–6]. On the other side, large databases allow one to detect uncommon reasons for revision and provide consistent comparisons and profile failures. This analysis was performed on large numbers (4318 modular implants), controlling some biases (in this case, evaluating the same bearings and the same cup) and providing cross-checked dependable outcomes [13].

The present report provided a comparison between modular and monoblock versions of the same stem: three different stem designs with the same bearings and the same cup were considered. Demographics were different among ST and DT cohorts: ST versions were preferentially implanted in younger and heavier men. It is likely that the choice of ST implants in these categories was dictated by the higher risk of neck failure in young and active patients [4–6]. Moreover, all the ST versions of these stems had standard and offset configurations, providing more solutions even for nonmodular stems [9–11].

In the three stem designs, the ST and DT versions achieved similar survival rates at 5 years. The survival rates of DT versions were far above 95% at 9 years. A similar comparison was provided by Duwelius et al., who compared ML Taper (Zimmer, Warsaw, USA) stems in DT and ST versions (594 and 284 implants, respectively) [3]. The authors achieved better radiographic results (leg length equality, offset reconstruction) in the DT cohort; however, no differences in terms of clinical outcomes and revision rates could be observed in the short term [3]. The authors cautioned against the indiscriminate use of modular implants due to the possible higher risk of failure [3]. Carothers et al. studied the radiographic outcomes of the two versions of the same stem, noticing no benefit of DT version; the authors reported 9/463 (2%) revisions, none due to neck breakage [7]. Thus, the conclusions were specular to those of Duwelius et al. [3, 7]. Schnurr et al. investigated the outcomes of Metha (Aesculap, Tuttlingen, Germany) stem, considering the results of ST (1165 implants) and DT (with titanium necks, 339 THAs, and cobalt–chrome necks, 259 implants) versions as collateral findings [14]. A higher revision rate in DT titanium neck cohort was evident (9.4%) in the short term, with fractures impacting on the cumulative revision rates (15, 4.4%). Other interesting outcomes were provided by the Australian registry on ABG II (Stryker, Mahwah, USA; 228 stems), ML Taper (Zimmer, Warsaw, USA; 2578 implants), and Metha (Aesculap, Tuttlingen, Germany; 84 THAs) stems [5]. The DT versions of the three stems demonstrated increased rates of revision, more than twice as high as ST rates at short-to-midterm follow-ups (and higher than 5% at 5 years) [5]. The authors did not stratify the DT versions by neck alloy, but it is likely that even the titanium-on-titanium alloy combinations did not achieve comparable performances with respect to ST versions [5]. It should be noticed that in the present report, which included 4318 DT implants, the highest DT version revision rate was 3.3% at 9 years, far below the above-described studies.

We did not observe any differences between ST and DT versions in terms of survivorship, when stem-focused endpoints were adopted. These outcomes were in contrast with the Metha case series provided by Schnurr et al.: the revisions needing stem removal were 1% in the ST version, 6.5% in the DT version with titanium neck, and 1.5% in the DT version with cobalt–chrome neck [14]. On the contrary, similar outcomes were highlighted in the paper by Di Martino et al. about the use of modular stems in development dysplasia of the hip [8]. As demonstrated in other papers, DT implants did not provide any consistent advantage in terms of dislocations and instability [15, 16]. This is a very relevant issue, as modular implants were developed with the aim to improve soft tissue tension and hip biomechanics: theoretically, a lower rate of dislocations would have ensued [17]. However, some beneficial effects on socket fixation were hypothesized, as a consequence of improved component positioning [8]. In the present report, no differences could be noticed.

The most striking drawback of titanium-on-titanium femoral modularity is neck breakage [1, 6, 14, 17, 18]. The report described three neck failures, all due to neck breakage, occurring in heavy men weighting more than 90 kg. The incidence of revisions due to neck breakage was much lower than in the Australian registry (0.0007% versus 0.2%), despite the inclusion of the sole ceramic-on-ceramic bearings (reported to cause more neck failures), and the different follow-ups (mid-term versus long-term, respectively) [5]. However, the outcomes of the present report are quite reassuring about neck breakage. Once again, the heavier men demonstrated to be unsuitable candidates for modularity due to the higher risk of neck breakage, as unanimously reported in the literature [5, 6, 17, 18].

While no differences were noted in large cohorts, modular implants may still provide some additional benefits even in cases with primary osteoarthritis. As specified by many authors, there are many sex- and age-dependent modifications of the standard femoral anatomy that conventional ST stems may fail to properly address [2, 19–22]. In these cases, inappropriate femoral reconstruction (namely reestablishment of offset and leg length) may lead to patients’ discomfort, limping, and dissatisfaction, even with medico-legal implications [20]. In this way, DT implants with validated outcomes may provide a viable option to improve the restoration of femoral biomechanics. Especially in case of outlier morphologies, the femur may be broached with a double version stem design, and ST or DT version may be chosen after an intraoperative trial checking of implant stability and soft tissue tension. This strategy may reduce the use of DT implants to a minimum, but at the same time DT solutions are available if conventional ST stems fail to restore the femoral morphology (between 20% and 40% of the cases) [2, 19–22].

In summary, DT versions did not achieve inferior 5-year outcomes in comparison with ST devices when the same stem design was adopted. The rate of neck failure was very low at mid-term. Considering the potential added value provided by consolidated implants with modular version in nonconventional anatomies, even in primary osteoarthritis, DT implants may still have a very selected role in THA for primary osteoarthritis.

## Data Availability

The datasets used and/or analyzed during the current study are available from the corresponding author and from RIPO (http://ripo.cineca.it/Reports.html), on reasonable request.
